# Prostate specific antigen and acinar density: a new dimension, the “Prostatocrit”

**DOI:** 10.1590/S1677-5538.IBJU.2016.0145

**Published:** 2017

**Authors:** Simon Robinson, Marc Laniado, Bruce Montgomery

**Affiliations:** 1 Frimley Park Foundation Trust, United Kingdom

**Keywords:** Acinar Cells, Prostatic Neoplasms, Diagnosis, PSA

## Abstract

**Background:**

Prostate-specific antigen densities have limited success in diagnosing prostate cancer. We emphasise the importance of the peripheral zone when considered with its cellular constituents, the “prostatocrit”.

**Objective:**

Using zonal volumes and asymmetry of glandular acini, we generate a peripheral zone acinar volume and density. With the ratio to the whole gland, we can better predict high grade and all grade cancer. We can model the gland into its acinar and stromal elements. This new “prostatocrit” model could offer more accurate nomograms for biopsy.

**Materials and Methods:**

674 patients underwent TRUS and biopsy. Whole gland and zonal volumes were recorded. We compared ratio and acinar volumes when added to a “clinic” model using traditional PSA density. Univariate logistic regression was used to find significant predictors for all and high grade cancer. Backwards multiple logistic regression was used to generate ROC curves comparing the new model to conventional density and PSA alone.

**Outcome and results:**

Prediction of all grades of prostate cancer: significant variables revealed four significant “prostatocrit” parameters: log peripheral zone acinar density; peripheral zone acinar volume/whole gland acinar volume; peripheral zone acinar density/whole gland volume; peripheral zone acinar density. Acinar model (AUC 0.774), clinic model (AUC 0.745) (P=0.0105). Prediction of high grade prostate cancer: peripheral zone acinar density (“prostatocrit”) was the only significant density predictor. Acinar model (AUC 0.811), clinic model (AUC 0.769) (P=0.0005).

**Conclusion:**

There is renewed use for ratio and “prostatocrit” density of the peripheral zone in predicting cancer. This outperforms all traditional density measurements.

## INTRODUCTION

PSA and derived densities, whole gland density, PSAD (1, 2) and the transition zone density PSATD (3), have a limited role in diagnosing cancer despite initial optimism. This is partly due to age related changes (4).We propose a new way of using the zones of the prostate taking into account their absolute volumes and the asymmetry in the amount of glandular acini within each (5-7). Hence the relative contribution of each zone, in terms of both epithelial acinar cells and their PSA production, to the entire gland. This highlights the contribution of the peripheral zone acinar volume (PZav) and its acinar density (PZad). We divide the serum PSA into the differing amounts of acini within zones of differing volumes.

The peripheral zone is an intrinsically more stable entity (in terms of volumes and relative amounts of stroma and acini). This allows an intuitive approach to use volumes with appropriate densities (not the arbitrary divising of entire PSA into the transition zone). It is these acinal cells that produce PSA, a marker of epithelial activity, and which undergo mitotic events causing cancer. Regardless of the nature of the nodule, there is always an increase in stroma relative to epithelium (8) and a diluting effect on glandular components. We have taken the overall glandular quantity in the entire gland to be 70%, most of which is concentrated in the peripheral zone (6, 8-10). This varies with age. The peripheral zone is far less variable in acini and stroma, and we extrapolate our other values from this constant entity.

We can better predict cancer of all grades, and more importantly, better predict high grade cancer than conventional densities. Finally, we propose that we will be able to model both the benign gland as it ages, in terms of each zone, its divisions into acinar and stromal elements, and further, that we can contrast this with the relative growth dynamics of the malignant gland.

## MATERIALS AND METHODS

Our study population included 672 patients admitted to a district general hospital, for transrectal ultrasound and biopsy, from 2007 to 2012, because of elevated PSA, anxiety or abnormal rectal exam. This was performed by one physician. The inner gland and the outer, peripheral zone were measured. 409 were benign and 263 malignant. We measured the whole gland volume (WGv) using the ellipsoid formula, then the inner gland (transition zone and central gland combined). We subtracted this from the WGv to yield the peripheral zone volume (PZv). We documented those with a positive family history, a first degree relative affected by prostate cancer. Also whether they had had a previous negative biopsy and whether the prostate felt suspicious of cancer or was clearly malignant (Table-1).

**Table 1:** Patient characteristics.

		BPH	PC
**Age years**	Mean	63.0	68.1
	Median	63.0	68.0
	Interquartile range	58-68	63-75
**PSA ng/mL**	Mean	8.1	16.4
	Median	6.6	8.5
	Interquartile range	4.6-9.1	5.8-13.3
**Whole gland volume cc**	Mean	57.6	48.0
	Median	51.0	40
	Interquartile range	35-71	29-59
**Transition zone volume cc**	Mean	29.8	22.4
	Median	24.0	16.0
	Interquartile range	14-39.5	9-29
**Peripheral zone volume cc**	Mean	27.7	25.7
	Median	24.0	24.0
	Interquartile range	18-34	17-31
**Previous negative biopsy**		93 (23%)	73 (28%)
**Family history**		48 (12%)	37 (14%)
**DRE**	Suspicious	102 (25%)	83 (32%)
	Cancer likely	4 (1%)	11 (4%)

We have accepted the established glandular, acinar component of the prostate of 70%. The WGv x 0.7 yields the whole gland acinar volume (WGav). By definition, 1–WGav yields the whole gland stromal volume (WGsv). The peripheral zone was attributed a percentage of 80%. 0.8 x PZv yields the peripheral zone acinar volume (PZav).

1-PZav yields the peripheral zone stromal volume (PZsv). The WGav – PZav yields the transition zone acinar volume (TZav).

**Figure 1:**
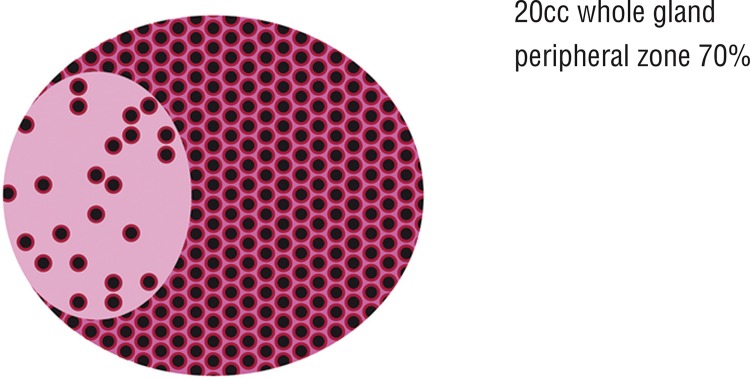
Normal gland 20cc schematic diagram. 70% PZ by volume 30% TZ by volume. PZ 80% acini 20% stroma (cells red, lumen black, stroma pink).

**Figure 2:**
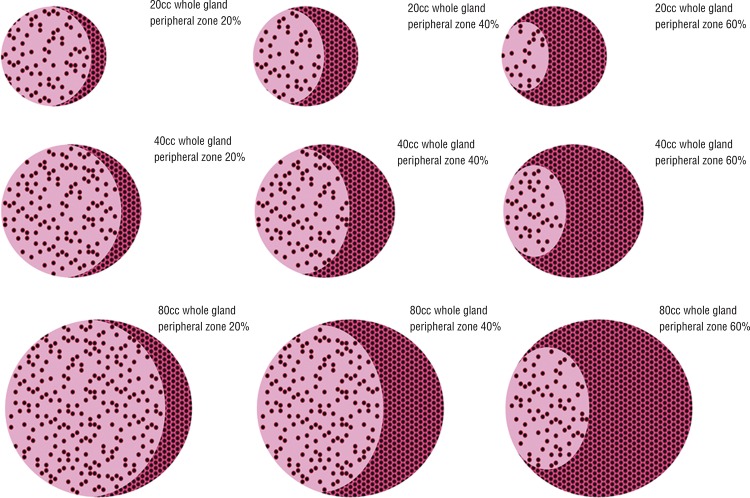
Prostate gland with variation in whole gland size and peripheral zone as 20, 40, 60% of whole gland volume.

1-TZav yields the transition zone stromal volume (TZsv) (Figure-1). Figure-2 demonstrates how the zones vary with a growing gland.

### Densities

The serum PSA is divided into WGav to yield the whole gland acinar density. The peripheral zone acinar density is calculated by multiplying the WGad times the ratio of the PZav/WGav.

The transition zone acinar density is WGad–PZad.

Formula:

0.7WGv = WGav

PSA/Wgav = WGad

0.8PZv = PZav

Wgav - Pzav = TZav

WGad x PZav/Wgav = PZad

WGad - Pzad = TZad

WGv - Wgav = WGsv

TZv - Tzav = TZsv

PZv - Pzav = PZsv

### Statistics

Univariate logistic regression was initially used to find significant predictors for all grades of prostate cancer and then for high grade (Gleason 7 and above). Backwards multiple logistic regression was used to generate ROC curves using the new model and comparing it to whole gland density, PSA density and to PSA alone using Medcalc statistical software.

## RESULTS

See Table 1

See Table 2

**Table 2:** All grade and high grade models.

All grade model	Variable	Coefficient	Standard error	P	Odds ratio	Confidence interval
	Age	0.089	0.01	<0.0001	1.09	1.067-1.11
	DRE = cancer	2.14	1.07	0.0466	8.5	1.03-70.0
	Family history positive	0.57	0.27	0.0382	1.7	1.03-3.08
	LnPZAd	0.58	0.16	0.0003	1.8	1.30-2.48
	Previous negative biopsy	-0.55	0.24	0.0218	0.57	0.35-0.92
	PZAv/WGAv	1.43	0.62	0.0223	4.18	1.22-14.28
	PZAd/WGv	60.1	26.23	0.0219	129E+24	6058-2.77E+048
	PZAd	-1.34	0.67	0.0470	0.26	0.06-0.98
High grade model	Age	0.06	0.014	<0.0001	1.07	1.04-1.10
	DRE suspicious	0.61	0.240	0.0103	1.85	1.15-2.96
	DRE cancer	3.49	1.105	0.0015	33.08	3.79-288.7
	Ln PZad	0.98	0.134	<0.0001	2.67	2.05-3.49
	Previous negative biopsy	-0.69	0.330	0.0346	0.49	0.26-0.95

Variables not included in all grade model; DRE suspicious; PSA; Race Caucasian; Race Afro-Caribbean; PZD; TZD; PSAD (WGD)

Variables not included in high grade model; family history; PSA; PZAD/WGV; PZAV/WGAV; PZD; Race Caucasian; Race Afro-Caribbean; TZD; PSAD (WGD)

See Table 3

**Table 3:** Positive and negative predictive values (PZad).

High grade prostate cancer						All grades of prostate cancer			

Mean PZad	No cancer	Cancer	Total	PPV%	NPV%	No cancer	Cancer	PPV%	NPV%
0.043	128	7	135	5	95	104	31	23	77
0.082	118	17	135	13	87	101	34	25	75
0.121	115	19	134	14	86	92	42	31	69
0.197	106	29	135	21	79	73	62	46	54
0.775	74	61	135	45	55	40	95	70	30

See Figure 3

**Figure 3:**
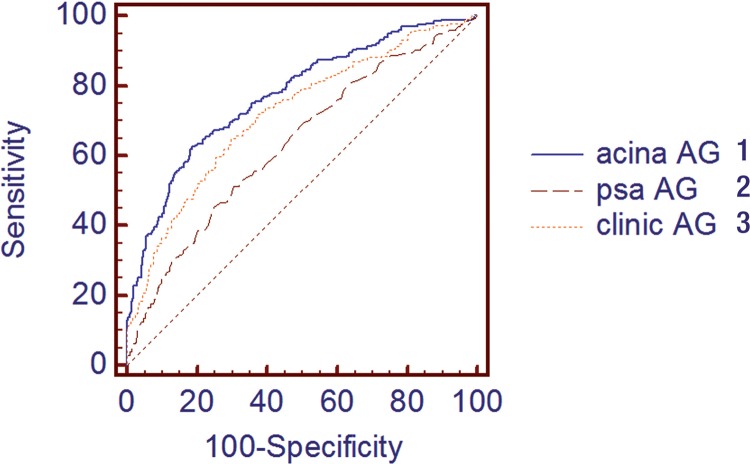
ROC for all grades (AG) of cancer. Comparing acinar ratio (Prostatocrit) model with clinic model and PSA alone

1 - Acinar prostatocrit model AUC 0.7742; 2 - PSA AUC 0.636 P = 0.0001; 3 - Clinic model (PSAD) AUC 0.745 P = 0.01053

See Figure 4

**Figure 4:**
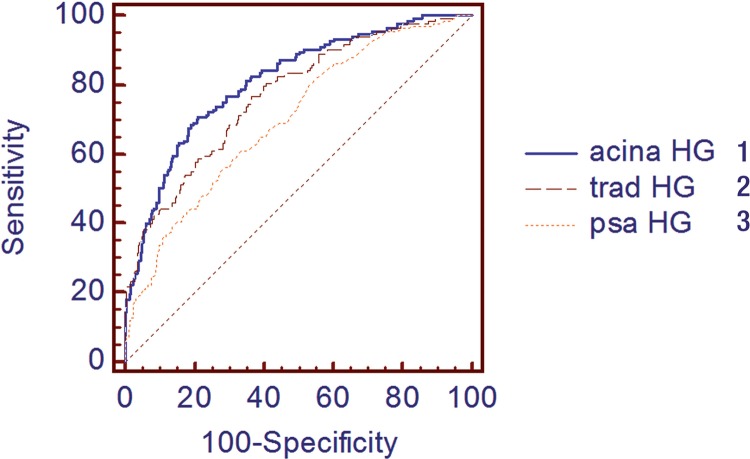
ROC for high grade cancer. Comparing prostatocrit model with clinic model and PSA alone.

1 - Acina prostatocrit model AUC 0.811; 2 - Clinic (PSAD) model AUC 0.782 P = 0.005; 3 - PSA model AUC 0.700 P = 0.0001

## DISCUSSION

### Developing the model

To tackle the problem of PSA and its variable zonal densities, we need to take into account the distribution of acini within the gland in the normal man and the age related changes of hyperplasia within nodules. The periphery was noted, in autopsies, to account for most of the glandular material (6) and the inner gland to account for most of the ducts and stroma. The asymmetry of gland distribution was further enforced when normal (autopsy) and enlarged (suprapubic prostatectomy) glands were compared (8). In the glands with benign prostatic hyperplasia, the glandular component was only 12% and this was mostly due to “dilution” by stromal growth. Growth rates were elucidated showing two phases, a rapid increase in growth from ten years old to thirty years then a slow gradual increase from thirty onwards. The normal stroma constituting about 50% of the mass and epithelium and acinal lumen the other 50%. Hyperplasia is associated with growth of both components to varying degrees (11). The ratio of stroma to epithelium, within adenomas, varies with symptoms (4.6) and without symptoms (2.6) (12). The stroma forms 62% of the symptomatic gland and epithelium 38%. The conclusion is that BPH is primarily a stromal process. Using enzymatic staining it was found the stroma to constitute 76% (12, 13) and that it is the ratio increase leading to symptoms.

That PSA is related to prostate size, the amount of epithelium and testosterone was well established. But size is not a good predictor of PSA because of the great variation in acini and whole gland volume with a range of stromal component (14). It was proposed that epithelium itself is not a good guide to the amount of PSA because the architecture of acini can be disrupted by disease processes both benign and malignant. Further, PSA secretion is androgen independent and this wanes with advancing years.

The predominance of epithelium within the PZ compared to TZ, yet the lack of correlation of PSA with the PZ and the greater correlation of PSA with TZ has been confirmed (7). Further, the variability of the TZ and the relative constancy of the PZ is documented. The confounding problem of PSA is that it appears to be highly dependent on the TZ rather than the PZ. The rate of BPH epithelium growth is x 9 the normal gland rate and stromal growth within BPH x 37 the normal rate (15). In regard to ratio, the converse is true for cancers.

The androgen receptor is present in both stroma and epithelium, but 5 alpha reductase is only present in stromal cells and that they have an inductive influence on the epithelium (16). The range of influencing factors are categorised as intrinsic and extrinsic and there is no simple relation to androgen levels (17). We see in our cohort that the BPH glands are bigger overall, with larger transition zones, a similar size of peripheral zone and that the patients are younger than those with malignant glands (Table-1).

Ever since PSA was first localized to the gland (18) its exact use in diagnosis has been hampered by a lack of cancer specificity (1).

The relation of serum PSA to the entire gland volume was proposed (2) to allow an individualised approach to patients with intermediate PSA levels. The relation of PSA to zones of the gland was established using correlation coefficients (4). When reviewing patients who had undergone radical prostatectomy and cystectomy, it is apparent that the peripheral zone is significantly larger than the transition zone. It was found the average ratio of TZ to PZ was 3:1. Part of the problem with assessing the gland is the great variability in the TZ ranging from 2-80% of the total volume (19, 20). Recent studies (21) fail to differentiate cancer using PSAD. Further refinement was attempted with free/total ratio and TZAD (22) with PSA in the range of 2.5-4ng/mL. However, this has great limitations with small glands. The relation of the whole volume to PSAD and TZAD was proposed (23) and improves specificity and can limit unnecessary biopsies. The role of the PSATD has been recently strengthened by showing it had the most predictive power in diagnosing cancer (24). However, its use in predicting stage (25) reveals the confounding influence of the TZ. Regarding the PZ, it is suggested that the cancer which arises here, does so because of more cells of epithelial origin that are undergoing cell division and potential cancerous changes will be more numerous here. It appears there is a difference in the ratio of the two zones in cancer patients compared to benign patients. The peripheral zone is intrinsically richer in the acini that make PSA (5). The whole issue of PSA density and zonal densities has been dominated by the adoption of the term transition zone density which divides the entire serum PSA into the volume occupied by the inner gland (3, 26, 27). This is an incorrect use mathematically. Practically, it can contribute to diagnosis (26, 7), but ideally, the relative contribution of the separate zones should be accounted for. This is intuitively confusing otherwise and we end up with total densities greater than the original. The division of total serum PSA into the TZ ignores the contribution of the PZ to serum PSA. The corresponding lack of use of the TZAD highlights this (28). The peripheral zone has had limited application so far, although it has proved useful in men on alpha reductase inhibitors (29).

To illustrate the problem, we compared the two approaches below for a 40cc gland with equal components of peripheral and transition zones and with a serum PSA of 4ng/mL.

Thus, WGd=0.1ng/mL/cc

### Traditional method

- TZ=20cc TZD=0.2ng/mL/cc
- PZ=20cc PZD=0.2ng/mL/cc
- Total density is now 0.4ng/mL/cc

### Prostatocrit method

- We have to take into account for the relative contributions of each zone
- The TD is 0.1 x 20/40=0.05ng/mL/cc
- The PD is 0.1 x 20/40=0.05ng/mL/cc total=0.1ng/mL/cc

We now have a density attributable to the zonal volume. This can be refined estimating the bulk of epithelium/acini within each zone.

We are aware of packed cell volume in haematology. The acini are equivalent to red cells, the stroma is equivalent to plasma and the PSA is equivalent to haemoglobin.

The gland is composed, overall, of 50-70% acini. Having accounted for different zonal volumes, we now need to account for the asymmetry of distribution of acini (Figures 1 and 2).

The peripheral zone is denser in acini by definition. Let us assume it is 80% acini. The transition zone must be less dense. For demonstration purposes, we choose 60% acini. The peripheral zone does not vary in its composition unlike the transition zone.

- For our 40cc gland with equal 20cc zones
- TZ=20cc x 0.6=12cc acinar volume
- PZ=20cc x 0.8=16cc acinar volume
- =28cc total acinar volume

This is the amount of epithelial tissue within the whole gland.

The density is derived by dividing the serum PSA into the relevant amount of acini.

- WGad=4/28=0.14ng/mL/cc of acini
- TZad=0.14 x 12/28=0.06ng/mL/cc
- PZad=0.14 x 16/28=0.08ng/mL/cc

Given that we can estimate the volume of acini within each zone, we can then by simple subtraction, estimate the amount of stroma, that is 1-acina %.

- TZsv=20-12=8cc
- PZsv=20-16=4cc
- 8+4=12cc stroma
- 12cc stroma + 28cc acini=40cc whole gland.

### Using the model

Univariate logistic regression for prediction of all grades of prostate cancer were used to test significant predictors in a multivariate logistic regression. There were four significant zonal predictors. All involved the peripheral zone. The log of the peripheral zone acinar density, the ratio of the peripheral zone acina volume to the whole gland acinar volume, the peripheral zone acinal density to the whole gland volume and the peripheral zone acinar density. None of the conventional zone densities, whole gland, transition or peripheral zone densities were included.

We compared ROC for this model with a “clinic” model using the same information: PSA, age, family history, previous negative biopsy, rectal examination and overall gland volume (Table-2 and Figure-3). We also compared PSA on its own. There was a significant improvement in the area under the curve from PSA alone, to the conventional clinic model, to the prostatocrit model using ratio and acinar density of the PZ in four different combinations, all significant and all superior to traditional density measurements.

Most importantly, of practical clinical significance, it differentiated high grade (Gleason 7 and above) cancers better than traditional parameters. The only significant predictor was the peripheral zone acina density. None of the traditional densities were significant (whole gland, transition and peripheral zone densities) (Table-2 and Figure-4). The influence of the peripheral zone acinar density is further illustrated in Table-3, which shows the increasing positive predictive value as this density increases and the converse, the decreasing negative predictive value.

### Limitations

TRUS is probably less accurate than MRI for measuring these volumes. These models will be improved with MRI and manual contouring of zones. We plan to do this in our next study. We also appreciate that other markers such as PCA3 and 4K could be included. The correlation of predicted acina density and final actual histological density will potentially strengthen this model.

Assumption of equal production of PSA in all types of acini in both periphery and transition zone. The formula is complex and will be part of a calculator, but this should not concern the physician.

### Strengths

This is an intuitive use of density and accounts for the relative constant amount of peripheral zone epithelium within an easily measured zone. The peripheral acinar zone was consistently significant in predicting all grade cancer and high grade cancer. It revealed four significant parameters all superior to traditional density measurements.

## CONCLUSIONS

When comparing the benign and malignant gland, the differences in ratio, with their acinal asymmetry, concentrated in the peripheral zone, enable the prostatocrit model to discriminate better between the two and hence who should have biopsies.

The absolute relation between zones, their acinal bulk and PSA production remains to be determined and may prove impractical, but this recognition of acinar contribution, may improve modelling of benign and cancerous disease, the response to drugs and need for surgery.

### Take home message

PSA, density, zones, acinal asymmetry provides a new dimension to the analysis of the prostate gland. This prostatocrit model better predicts high grade cancer, all grades of cancer and it will help describe natural benign growth of the separate zones.
